# City-Planning Against Natural Disasters: Lessons from Japan

**DOI:** 10.31662/jmaj.2025-0226

**Published:** 2025-12-05

**Authors:** Misa Tomono, Soichiro Saeki

**Affiliations:** 1 Department of Emergency Medicine and Critical Care, National Center for Global Health and Medicine, Japan Institute for Health Security, Tokyo, Japan; 2 Division of Public Health, Department of Social Medicine, Graduate School of Medicine, The University of Osaka, Osaka, Japan

**Keywords:** natural disasters, earthquakes, international collaboration, disaster medicine, global health, migrant health, travel health

## Abstract

On March 28, 2025, a 7.7-magnitude earthquake struck Myanmar, causing severe damage in Bangkok, including the collapse of a high-rise building. This incident revealed the structural vulnerabilities of rapidly urbanizing cities such as Bangkok. First, adopting earthquake-resistant building codes, similar to Japan’s “Building Standard Act,” is essential in areas with high population density and skyscrapers. Second, Bangkok, as the world’s top tourist city, faces challenges in crisis communication due to language barriers. A government-managed mobile application providing multilingual alerts, shelter information, and embassy contacts is proposed to assist foreign visitors. Strengthening both infrastructure and communication systems is critical to improving resilience and protecting residents and tourists during future disasters.

## Introduction: The March 28 Earthquake

On March 28, 2025, a powerful 7.7-magnitude earthquake struck central Myanmar near Mandalay ^(1)^, the nation’s second-largest city. As of April 4, reports indicate that the death toll has exceeded 2,000 ^(2)^. The tremors reverberated across neighboring countries, notably causing significant damage in Bangkok, Thailand, where the collapse of a high-rise building led to 13 deaths ^(2)^.

When a crisis occurs, especially in densely populated urban centers such as Bangkok, the impact is often amplified due to unique characteristics such as high population density, complex logistics, and greater dependency on centralized services. We aim to highlight the structural and systemic vulnerabilities specific to urban cities, using Bangkok as a representative case, and propose strategic interventions to mitigate risks in future crises. Given Japan’s long-standing experience in coping with frequent and severe earthquakes, this Opinion particularly focuses on Japanese examples as illustrative cases, with the aim of offering insights that may be applied to other disaster-prone urban settings.

## Structural Vulnerability in High-Density Cities

First, urban cities have a unique vulnerability to high population density and skyscrapers with poor construction techniques. As third-world countries experience economic growth and urbanization, the frequency and severity of earthquakes in urban areas worldwide have increased ^(3), (4), (5)^. Bangkok has experienced rapid economic expansion since the 2000s, leading to a surge in construction projects. The recent collapse of a high-rise building under construction underscores the potential dangers associated with accelerated urbanization in the absence of stringent seismic design protocols. In densely populated cities, such as Bangkok, the collapse of high-rise buildings can obstruct evacuation routes and limit access to safe zones. Although there were no specific reports of roads being blocked due to building collapses in this incident, the potential for such scenarios remains alarming.

Thailand must adopt comprehensive earthquake-resistant building codes to mitigate such risks by drawing on international best practices. Japan offers a compelling model through its “Building Standard Act”, which has undergone multiple revisions following major earthquakes. After the 1978 Miyagi-ken-Oki earthquake, the Building Standard Act was revised in 1981 to introduce the “New Seismic Design Method”, requiring buildings to withstand *shindo* (seismic intensity) 6-7 without structural collapse ^(6)^. Following the 1995 Great Hanshin-Awaji Earthquake, further revisions were introduced, including mandatory soil condition surveys, enhanced column-beam joint standards, and stricter evaluation of structural irregularities ^(6)^. In response to the current earthquake, the Japanese government sent a team of architectural experts from the Japan International Cooperation Agency and the Ministry of Land, Infrastructure, Transport, and Tourism of Japan in April to collaborate with Thai authorities ^(7)^. This initiative offers a pivotal opportunity for bilateral cooperation in codifying earthquake-resistant architectural standards suited to the growth of Thailand’s urban landscape.

## Tourism and Crisis Communication

Bangkok is the world’s top tourism city in 2024 after welcoming a record-breaking 32.4 million visitors ^(8)^. Effective communication during crises is crucial, especially considering language barriers ^(9)^ and the unfamiliarity of tourists with region-specific natural disasters such as earthquakes or typhoons. Given this background, several studies have categorized tourists as vulnerable groups in disaster situations ^(10), (11)^. To address these challenges, Thailand should implement multilingual disaster communication platforms tailored to foreign visitors, ideally in the form of an official mobile app (http://mobile.tourismthailand.org/) managed by the Thai government or the Tourism Authority of Thailand. The application should serve as a central hub for real-time alerts and guidance, including

1)Earthquake and aftershock alerts,

2)Locations and capacities of evacuation shelters,

3)Embassy contact information by nationality,

4)Safe transportation routes and airport status.

Japan’s “Safety tips” app (https://www.jnto.go.jp/safety-tips/eng/app.html) offers a viable template, delivering push alerts in multiple languages and safety instructions. In addition, mainstream and social media platforms should coordinate reliable updates in multiple languages using plain language and visual aids. Emergency preparedness training should be institutionalized in the hotel and hospitality sectors to empower staff to assist international guests during crises. Earthquake risk communication is not confined to national borders. With increasing global mobility, tourists from low-risk countries frequently visit high-risk destinations, often without adequate disaster preparedness. Thus, integrating multilingual alerts and cross-border cooperation is essential for protecting transient populations.

## Conclusion: Building Back Better

In conclusion, the recent earthquake serves as a stark reminder of the inherent vulnerabilities of urban systems to natural disasters. Addressing these challenges requires a multidimensional strategy encompassing robust infrastructure, proactive urban planning, and inclusive communication strategies that extend to transient and non-native populations. By embracing these measures, Bangkok and cities facing similar risks can improve their resilience and the well-being of residents and visitors in future crises.

## Article Information

### Acknowledgments

The authors thank their colleagues for helpful discussions on this topic. The authors acknowledge the use of Grammarly (Grammarly Inc. San Francisco, USA) for primary language editing. The views expressed in this manuscript are those of the authors and do not necessarily represent those of the authors’ institutions.

### Author Contributions

Misa Tomono prepared the first draft of the manuscript. Soichiro Saeki critically reviewed the manuscript. All authors have read and approved the final version of this manuscript. Artificial intelligence technology was used for the language editing process, and the authors reviewed the final content. The authors’ institution played no role in the conceptualization of this manuscript.

### Conflicts of Interest

None

### IRB Approval Code and Name of the Institution

Not applicable.

### ORCID iD

Misa Tomono 0000-0002-1905-0118 https://orcid.org/0000-0002-1905-0118

Soichiro Saeki 0000-0002-3288-423 https://orcid.org/0000-0002-3288-423X
